# The Role of Human Microbiota in Myasthenia Gravis: A Narrative Review

**DOI:** 10.3390/neurolint15010026

**Published:** 2023-03-10

**Authors:** Giuseppe Schirò, Salvatore Iacono, Carmela Rita Balistreri

**Affiliations:** 1 Neurology Unit, Department of Biomedicine, Neuroscience and Advanced Diagnostics (BiND), University of Palermo, 90127 Palermo, Italy; 2 Cellular and Molecular Laboratory, Department of Biomedicine, Neuroscience and Advanced Diagnostics (BiND), University of Palermo, 90127 Palermo, Italy

**Keywords:** myasthenia gravis, gut–brain axis, inflammation, oral microbiota, gut microbiota, Western diet, cognitive decline, depression, anxiety, probiotics

## Abstract

Myasthenia gravis (MG) is an autoimmune neuromuscular disease characterized by fluctuating weakness of the skeletal muscles. Although antibodies against the neuromuscular junction components are recognized, the MG pathogenesis remains unclear, even if with a well-known multifactorial character. However, the perturbations of human microbiota have been recently suggested to contribute to MG pathogenesis and clinical course. Accordingly, some products derived from commensal flora have been demonstrated to have anti-inflammatory effects, while other have been shown to possess pro-inflammatory properties. In addition, patients with MG when compared with age-matched controls showed a distinctive composition in the oral and gut microbiota, with a typical increase in *Streptococcus* and *Bacteroides* and a reduction in *Clostridia* as well as short-chain fatty acid reduction. Moreover, restoring the gut microbiota perturbation has been evidenced after the administration of probiotics followed by an improvement of symptoms in MG cases. To highlight the role of the oral and gut microbiota in MG pathogenesis and clinical course, here, the current evidence has been summarized and reviewed.

## 1. Human Microbiota: Composition and Functions

The human microbiota embodies the whole population of microorganisms present in the human body and is mainly represented by the gut microbiota. Bacteria are the main components of the gut microbiota, but protists, archaea and virus represent the other components, even if present in a smaller amount [1]. They are principally represented by Firmicutes, *Bacteroidetes* and *Actinobacteria*, which account for more than 90% of the gut bacteria and are essential for maintaining the homeostasis of the entire intestinal bacterial flora [2]. Despite the myriads of possible combinations, it has been shown the existence of a limited number of balanced host–microbial symbiotic states. Accordingly, the existence of three different enterotypes has been observed, each one resulting from the variation in the levels of one of following genera: *Bacteroides*, *Prevotella* and *Ruminococcus* [1]. The integrity of the gut microbiota guarantees many physiological processes of the host such as intestinal barrier function, digestion, metabolism of dietary elements and vitamins biosynthesis. Thus, many are the functions of human microbiota; in particular, the immunomodulatory function and the bidirectional interaction with the central nervous system (CNS) have reached great consideration in the last decades. *Clostridia* accounts for 95% of the *Firmicutes* phylum resident in gut microbiota, representing the main producers of short-chain fatty acids (SCFAs) through the fermentation of proteins and carbohydrates. Of note, SCFAs (e.g., propionate and butyrate) support the differentiation of naive CD4+ T cells into Foxp3+ CD4+ T regulatory cells (Tregs) by stimulating acetylation of the H3 histone at the promoter of the Foxp3 gene regulating the anti-inflammatory responses through G-coupled protein receptor 43 [3]. Moreover, the gut microbiota–immune system interplay also regulates the production of immunoglobulins, in particular mucosal IgA. In fact, if the bacterial presence decreases, the local antibody response also decreases. This probably depends on the fact that the production of IgA induces the microbiota to penetrate Peyer’s patches, providing a positive reinforcement to the generation of IgA [4]. In addition, circulating SCFAs may regulate the permeability of endothelial tight junctions enhancing the integrity of the blood brain barrier (BBB). The relationship between gut microbiota and CNS is known as the microbiota gut–brain (MGB) axis, a concept first proposed in 2012 [5]. This closed relationship has been demonstrated to be mediated also by neuroanatomical structures existent between the brain and gut, and via intestinal nerves located in the intestinal wall [5]. In this view, the intestinal information can be transmitted to the brain by the vagus nerve, with a response via its descending branch which regulates, in turn, intestinal activities. In addition, another component of the MGB axis is the neuroendocrine axis, represented by the hypothalamic–pituitary–adrenal (HPA) axis. It allows the regulation of the major pathway in gut–brain communication under stress conditions [6]. The HPA determines variations in gut microbial composition and functions by its activation. Dysfunctions in the HPA have been demonstrated to have a crucial role in the pathogenesis of neuropsychiatric diseases. Precisely, the HPA mediates the activation of the inflammatory signaling pathway with the consequent release of inflammatory mediators, such as tumor necrosis factor α (TNF-α), interferon-γ (IFN-γ) and interleukin 6 (IL-6) [7]. In turn, these mediators can contribute to the destruction of BBB integrity and development of brain diseases, via systemic circulation and by damaging the gut mucosal barrier. In addition, inflammatory-induced HPA response also influences the secretion of glucocorticoids [8], which in turn activate intestinal function and the production of pro-inflammatory factors [9]. This vicious circle also causes the activation of enteric immune cells, such as T helper (Th) 17 and natural killer cells that can translocate into the brain and cause neuroinflammation [10]. Neuroinflammation, in turn, also alters the gut microbial composition, which ulteriorly stimulates enteric immune cells and microbiota-derived metabolites acting as regulators in this bidirectional via inflammatory signals.

Because of the immunoregulatory function of gut microbiota, the alterations of commensal microbiota (i.e., dysbiosis), recently, have attracted an increasing interest regarding its pathogenetic role in immune-mediated diseases. Indeed, dysbiosis is characterized by the alteration of host–microbe interaction, and it has been associated with a low-grade inflammation, metabolic syndrome, infections of the gastrointestinal tract and inflammatory bowel disease [11,12,13,14]. Of note, the reduced *Firmicutes*/*Bacteroidetes* ratio (F/B ratio) due to the increase in the Bacteroidetes phylum has been found to be associated with the pro-inflammatory shift of the gut microbiota in autoimmune diseases such as systemic lupus erythematosus, systemic sclerosis, Sjogren’s syndrome, antiphospholipid antibody syndrome, multiple sclerosis (MS) and myasthenia gravis (MG), while F/B ratio alteration is conflicting in obesity wherein some authors found a reduction while other found an increase [15,16,17,18]. Although the gut microbiota has reached a great consideration, the oral microbiota has been neglected. It has been estimated that 500 to 700 different species inhabit the oral cavity and among these the species Streptococcus mitis is the most abundant [19,20]. However, the composition of oral microbiota is extremely variable depending on oral habitats (e.g., tongue, cheeks, teeth) as well as on the presence of exogenous pathogens and/or the overgrowth of previously existing resident oral microbiota [19,21]. Although the role of oral microbiota in MG is almost unexplored, its involvement in the pathogenesis of autoimmune disease is attractive. Indeed, the increase in the *Staphylococcus*, *Actinomyces* and *Bacteroides* genera and the reduction in *Lactobacillus* have been recently found in the oral cavity of patients with MS [22]. MG is a neuromuscular autoimmune disease characterized by the immune-mediated destruction of the neuromuscular junction (NMJ). Although antibodies against the neuromuscular junction components are recognized, the MG pathogenesis remains unclear and it is probably multifactorial [23]. Based on the emerging evidence on the roles of microbiota in the pathogenesis of other autoimmune disease such as MS, the perturbations of human microbiota have been recently suggested to contribute to MG pathogenesis and clinical course. Accordingly, some products derived from commensal flora have been demonstrated to have anti-inflammatory effects, while other have been shown to possess pro-inflammatory properties.

Here, we seek to shed light on the relationship between oral and gut microbiota and MG pathogenesis or disease course, by summarizing and reviewing current evidence. In the next chapters, the actual studies exploring the perturbations of oral and gut microbiota in MG and its pathogenetic implications are reported.

## 2. Factors Influencing Microbiota

Factors influencing the composition and activity of the gut microbiota can alter the balance that exists between the host and the microbiota by compromising its functions. In particular, diet, aging, exercise and antibiotics can affect gut microbiota composition [24]. Diet is one of the main factors capable of shaping the composition and functions of the gut microbiota and the different types of diet can mediate beneficial or negative effects. Indeed, the Mediterranean diet (MD) promotes microbial diversity and expands beneficial bacterial taxa: (1) fruits and vegetables are able to reduce the growth of potentially harmful bacteria; (2) polyunsaturated fatty acids (PUFAs) have been shown to reduce the *Firmicutes*/*Bacteroidetes* ratio increasing SCFA-producing bacteria (e.g., *Bifidobacterium*, *Lachnospira*, *Roseburia* and *Lactobacillus*) enhancing the intestinal barrier functions [25,26]. The beneficial effect of MD on waist circumference, glucose and lipid metabolism is well known. Furthermore, MD is able to reduce chronic systemic inflammation influencing thus the clinical course of chronic autoimmune disease [16]. Other diets such as low carbohydrates diets (e.g., ketogenic diet, paleolithic diets, etc.), gluten-free diet, fasting-mimicking diets and whole food plant-based diet have been associated with a reduced pro-inflammatory systemic state but their role in MG is almost unexplored or inconclusive [27].

**Figure 1:**
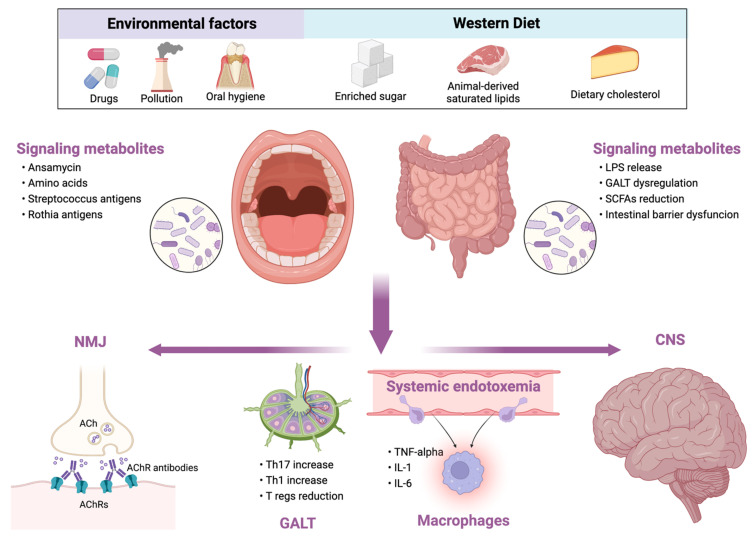
Relationship between external influencing factors and human microbiota and its consequences in myasthenia gravis. LPS, lipopolysaccharide; GALT, gut-associated lymphoid tissue; SCFAs, short-chain fatty acids; NMJ, neuromuscular junction; ACh, acetylcholine; AChRs, acetylcholine receptors; Th, T helper cell; Tregs, T regulatory cells; TNF, tumor necrosis factor; IL, interleukin; CNS, central nervous system.

Conversely, diets rich in animal-based proteins and saturated fatty acids (e.g., Western diet) reduce microbial diversity, increase the abundance of the potentially pathogenic and pro-inflammatory bacteria (Figure 1) [26]. Similar changes occur in the elderly. During aging, a loss of microbial diversity and a shift toward a population characterized mainly by Bacteroidetes was observed [28]. Although there are no studies exploring the role of MD in myasthenia gravis, given the beneficial effects of MD, we support the use of MD in these patients rather than other potentially harmful diets.

Exercise also exerts beneficial effects on the microbiota. In both animal models and humans, physical exercise can increase butyrate-producing bacteria. In particular, the use of running wheels by mice increased Firmicutes and improved cognition [29], while treadmill running was able to reduce Bacteroides with subsequent cognition improvement and neuroinflammation reduction [30]. Resistance-based training was able to promote the production of SCFAs as well as reduce potentially harmful bacteria such as Bacteroides and instead increase Faecalibacterium and Lachnospira [31].

The excessive use of antibiotics can induce a state of dysbiosis which is rapid to establish and long to be reversed, characterized by reduced colonization of the colon by bacteria resistant to invasion by other pathogens and by a decline in the taxonomic diversity with loss of functional differentiation. Moreover, it has been hypothesized that the use of antibiotics may also favor the development and maintenance of inflammatory bowel disease [32]. Moreover, the increased incidence of autoimmune diseases has increased in parallel with the increased use of antibiotics. Incorrect use of antibiotics could alter the intestinal immune response in two ways: firstly, by reducing the bacteria that are directly able to mediate an anti-inflammatory response and to stimulate the production of Tregs cells, such as the *Lactobacillus* and *Bifidobacterium*; secondly, by reducing the production of SCFAs capable of regulating the immune response at a peripheral level [33]. Education of the immune host system by the gut microbiota is fundamental for establishment of self-tolerance and response to the toxic antigens. In fact, intestinal Tregs are induced by the colonization of the colic mucosa by commensal bacteria such as *Clostridium* clusters IV, XIVa, XVIII and *Bacteroides fragilis*. Apart from nutritional factors and drugs, pathogenetic colonization may also alter microbiota functioning. Indeed, *Listeria monocytogenes* and *Toxoplasma gondii* are able to alter the T cell–gut microbiota interaction, resulting in an immune response driven by Th1 cells. It is possible that factors such as diet or antibiotics act as disruptors of microbiota, secondarily inducing alterations of the immune response. Priming of the immune response in the presence of gut dysbiosis could therefore result in the acquisition of pathogenic properties by the immune system and contribute to the induction of autoimmune diseases. Studies exploring the alteration of oral and gut microbiota in patients with MG are limited, although they are increasing.

## 3. Gut Dysbiosis

Dysbiosis may be sustained by detrimental dietary habits such as a Western diet and low vitamin D intake, as well as by proton pump inhibitors (PPIs) and consumption of other drugs [34,35,36]. The reduction in *Clostridia* and the subsequent reduction in SCFAs in patients with MG has been hypothesized as one of the pathogenetic mechanisms contributing to MG onset [15,17,18,37]. In particular, the reduction in *Clostridia* clusters IV/XIVa may be the reason for Foxp3+ CD4+ Tregs reduction in patients with MG. Consequently, the reduction in Foxp3+ CD4+ Tregs does not balance the activity of autoreactive lymphocytes that enhance the production of autoantibodies such as anti-AChRs antibodies [38]. A polymerase chain reaction analysis of fecal samples conducted by Moris et al. also showed the alteration of intestinal microbiota in MG patients compared with healthy controls. In particular, they found: (1) reduction in the phyla *Verrucomicrobia* and *Actinobacteria*; (2) increase in *Bacteroidetes* phylum; (3) increase in *Bacteroides* and *Desulfovibrio* genera; (4) reduction in *Bifidobacterium* genus, mainly sustained by the reduction in Bifidobacterium subsp. longum and Bifidobacterium adolescentis [39]. Another study showed that the *Faecalibacterium* genus (belonging to *Firmicutes* phylum) was abundant in fresh fecal samples from patients with MG when compared to patients with non-inflammatory diseases, but not compared with patients with chronic inflammatory demyelinating polyradiculoneuropathy [40]. The increase in Faecalibacterium in fecal samples of patients with MG appeared to be contrasting with a previous finding showing their reduction, particularly in *Faecalibacterium prausnitzii* [37]. However, the majority of studies on the alterations of the gut microbiota in MG concerned adult patients. Accordingly, in the literature there is currently available only a study in pediatric patients conducted by P. Liu and colleagues, who analyzed the fecal microbiota in 53 pediatric MG patients. The authors found that the increase in the *Cyanobacteria* phylum and other genera (i.e., *Prevotella*, *Ralstonia*, *Sutterella*, *Methyloversatilis*, *Providencia* and *Vibrio*) was able to identify patients with MG rather than healthy controls suggesting that microbial markers may serve as diagnostic biomarkers of MG [41]. In particular, at the species level, *Clostridium bartlettii*, *Bilophila wadsworthia* and *Bacteroides dorei* were found in healthy controls while *P*. *copri* and *Bacteroides massiliensis* were found in MG patients. In addition, they found that these alterations were associated with a reduction in serum SCFAs (i.e., butyrate and isobutyrate) [41]. Disruption of gut microbiota homeostasis, namely dysbiosis, is characterized by the reduction in beneficial bacteria and a parallel increase in pathogenetic bacteria leading to increased permeabilization of the intestinal wall and an imbalance between Foxp3+ CD4+ cells and T helper 17 (Th17) [42,43,44,45]. Foxp3+ CD4+ cells are a group of Tregs lymphocytes able to regulate immunotolerance as well as prevent autoimmune diseases [46]. The alterations of these cells are well known in the pathogenesis of MS [47]. In MG patients, especially in those with antibodies against AChRs, a numerical and functional reduction in Tregs has been found with a parallel increase in pro-inflammatory Th17 cells wherein it was associated with higher disease severity [48,49]. Of note, Tregs are most abundant in the gut-associated lymphoid tissue (GALT); thus, it is not surprising that the microbiota and its alterations have been described as a starting or aggravating factor in many autoimmune diseases such as MS, rheumatoid arthritis, inflammatory bowel disease, systemic lupus erythematosus, allergic disorders and MG [50,51,52,53,54].

There are many mechanisms through which dysbiosis affects the immune system and subsequently the pro- and anti-inflammatory balance. First, the death of the components of the *Bacteroidetes* phylum, prevalently including Gram-negative bacteria displaying lipopolysaccharide (LPS) on their surface, determines the release of LPS and systemic endotoxemia subsequently causing macrophage activation and pro-inflammatory cytokines production (e.g., IL-1, IL-6, TNFα) [55]. Second, dysbiosis also affects the production of SCFAs, particularly of propionate and butyrate, having immunoregulatory function such as the increase in Foxp3+ CD4+ Tregs [56]. Accordingly, the reduction in *Firmicutes* alters the production of butyrate, being Gram-positive bacteria including the genera *Lactobacilli*, *Bacilli*, *Clostridia*, *Enterococci* and *Ruminicocci*. Butyrate has the principal function of regulating intestinal homeostasis by reducing gut permeability with the increase in tight junctions and decreasing the intestinal inflammatory reactivity [57]. Likewise, *Clostridia*, particularly clusters IV and XIVa, are able to induce the differentiation of Tregs rather than other pro-inflammatory subsets also promoting their accumulation in GALT [39,58,59]. A lower presence of *Clostridia* has been found in the gut microbiota of patients with MG compared with controls and this is probably one of the causes of reduced Tregs in patients with MG. Finally, dysbiosis has been involved in the upregulation of genes involved in antigen presentation to B and T cells, and activation of the complement and coagulation cascade leading to intestinal barrier dysfunction [60].

Thus, dysbiosis would cause a reduction in anti-inflammatory metabolites such as propionate and butyrate with the subsequent Tregs reduction and Th17 expansion, as well as it promotes systemic inflammation and intestinal barrier dysfunction. In this view, further longitudinal studies are needed to better assess the role of dysbiosis in the pathogenesis of autoimmune diseases and, particularly, of MG. Indeed, given the acquired nature of the dysbiosis, the restoration of gut microbiota though probiotics or lifestyle interventions (i.e., alimentary habits, physical activity) might prevent MG onset or mitigate disease severity.

## 4. Oral Dysbiosis

Although the oral microbiota has been neglected so far, the role of oral dysbiosis in the pathogenesis of autoimmune or neurodegenerative diseases is attracting. First, oral dysbiosis can cause periodontitis, endodontic infections and dental caries which are all together connected with systemic inflammation. There are several studies relating oral dysbiosis and periodontitis with the pathogenesis of Alzheimer’s disease, Parkinson’s disease and MS [61,62]. Second, gut microbes originate, in part, from the oral cavity, and the alterations of oral microbiota may affect the gut microbiota as a consequence [37,63,64]. However, the relationship between oral microbiota and neurological disease derives from the increased release of pro-inflammatory cytokines in the case of dysbiosis leading to systemic inflammation [61]. To date, only one study explored the oral microbiota in MG patients.

By gene sequencing, Huang et al. showed a perturbation of the oral microbiota in 20 newly diagnosed MG patients, all positive for AChRs antibodies. In particular, the abundance of the phyla *Firmicutes* and *Actinobacteria* was increased in patients with MG, while *Proteobacteria* were the predominant phylum in healthy controls. Furthermore, they evidenced that bacteria of the genera *Streptococcus*, *Rothia*, *Lachnoanaerobaculum* and *Oribacterium* were increased in MG patients while the proportions of *Neisseria*, *Haemophilus* and *Treponema* were significantly decreased when compared with healthy controls [65]. Oral abundance of *Firmicutes* was, in contrast with their reduction in the gut microbiota, reported in other studies. This difference may be related to the different distribution of microbes in oral and gut microbiota as well as with different dietary habits or environmental factors. On the contrary, the higher abundance of *Streptococcus* in the oral cavity is consistent with the profile of intestinal flora in MG patients [37]. There are several pathogenetic mechanisms through which oral dysbiosis may affect the MG onset or disease course: (1) *Streptococcus* may modulate peroxisome proliferator-activated receptor γ affecting the balance between Tregs and Th17 [66,67]; (2) *Rothia* antigens can activate lymphocytes as well as induce macrophage activation and TNF-α production which can directly destroy AChRs or it may induce the differentiation and the growth of B cells allowing AChR antibody production [68,69]; (3) oral dysbiosis may alter the biosynthesis of ansamycin and some amino acids (e.g., valine, leucine, isoleucine, glutamine). Huang et al. hypothesized that the increased ansamycin, which is a macrolide, may interfere with neuromuscular transmission, unmasking MG symptoms according to the well-known effects of macrolides on the NMJ [65,70]. On the other hand, because some metabolites have immunomodulating properties, the altered amino acid metabolism may play a further role in the MG pathogenesis.

However, because the composition of oral flora depends on many factors, the relationship between oral microbiota and MG is probably multifaced. First, the primary alteration of oral microbiota leading to pro-inflammatory changes may play a role in the pathogenesis of MG. Second, patients with MG may have a secondary alteration of oral microbiota considering the numerous drugs that they consume such as corticosteroids, PPIs, pyridostigmine and immunosuppressants that may alter the host–microbe interaction in the oral cavity. Finally, several factors influence oral microbiota composition such as oral hygiene, dietary habits, smoking and others, making it extremely difficult to establish the specific host–microbial symbiotic states predicting MG risk or disease severity. The changes in gut and oral microbiota in MG patients are reported in Table 1.

## 5. Clinical and Therapeutics Implications

MG is characterized by fluctuating skeletal muscle weakness ranging from fatigability to dyspnea and respiratory failure due to the involvement of respiratory muscles [23]. The clinical manifestations result from the immune-mediated attack of the NMJ caused by AChRs, muscle-specific kinase or anti-low-density lipoprotein receptor-related protein 4 antibodies [74,75]. The MG pathogenesis is multifactorial probably resulting from a complex gene–environment interaction. As well, many factors may aggravate the MG clinical course reducing the patient’s quality of life such as drugs, hot temperature, systemic infections and comorbidities (e.g., autoimmune, diabetes mellitus, obesity, anxiety, depression, etc.) [76,77,78]. Comorbidities may arise from the same pathogenetic mechanisms underlying MG (e.g., autoantibodies) or they may be caused by MG treatment (e.g., iatrogenic), ageing and other factors. Recently, mild cognitive impairment and behavioral alterations (i.e., depression, sleep disturbances) have been shown to be common in patients with MG wherein they were associated with higher disease severity and disability [79]. Apart from autoimmune diseases, gut dysbiosis (e.g., lower presence of *Firmicutes* and an increase in *Bacteroidetes*) has been linked to cognitive impairment and psychiatric diseases [80]. It has been shown that colonization of mice with fecal microbiota from subjects with MG was able to induce anxiety-like behavior in female mice. In addition, the anxiety phenotype was reverted by colonizing mice with fecal microbiota from both MG patients and healthy individuals (i.e., co-colonization). Furthermore, 146 different metabolites in the gut and in three brain areas (i.e., hippocampus, striatum and prefrontal cortex) were identified in mice colonized with fecal microbiota of MG patients which was reversed by co-colonization [80]. These findings suggest that the perturbation of gut microbiota may be involved in the progress of anxiety-like behavior in MG. In addition, since the involvement of the hippocampus and prefrontal cortex, we may speculate that cognitive decline may be, in part, related with gut–brain axis perturbation in MG.

In this view, cognitive decline, psychiatric comorbidities and MG may, in part, represent the epiphenomenon of the systemic pro-inflammatory shift resulting from dysbiosis and the subsequent alteration of the gut–brain axis (Figure 1). However, the impact of microbiota in MG clinical course and comorbidity should be better clarified in the future.

Probiotics are live bacteria that may help to restore the host–microbiota balance. The role of probiotics in diverse diseases has been explored by several studies but little is actual known on the role of probiotics in MG. Apart from the host–microbiota rebalance, probiotics have shown the ability to interact directly with GALT, therefore the effect of probiotics in autoimmune diseases is intriguing [52,81,82]. The use of various probiotics to rebalance microbiota disruption in MG has been attempted in some studies performed in both humans and animal models through experimental autoimmune MG (EAMG) [83]. Chae et al. showed that the five-times-per-week administration of IRT5, an equal ratio mixture of five probiotics including *Streptococcus thermophilus*, *Lactobacillus reuteri*, *Bifidobacterium bifidium*, *Lactobacillus acidophilus* and *Lactobacillus case*, starting two weeks before EAMG induction, was able to improve EAMG progression through the inhibition of AChR-reactive lymphocyte proliferation, lower inflammatory cytokine expression and lower anti-AChR antibody production. However, no therapeutic effect was observed when probiotics were administered during the acute phase of EAMG [84]. More recently, Rinaldi et al. showed that the early administration of *Bifidobacteria* (i.e., *B. animalis subsp*. *lactis* BB12 and *B. animalis subsp. lactis* LMG S-28195) and *Lactobacilli* (*L. crispatus* LMG P-23257 and *L. rhamnosus* ATCC 53103) at EAMG onset was able to improve disease symptoms with the subsequent reduction in AChRs antibodies and the increase in immunomodulatory markers (e.g., Foxp3, transforming growth factor beta, cytotoxic T lymphocyte antigen 4) in draining lymph nodes and spleen in rats [82]. These immunoregulatory effects were observed after two and four weeks in the case of *Lactobacilli* and *Bifidobacteria* administration, respectively. However, the effects of the two *Bifidobacteria* were stronger than the *Lactobacilli* combination [52].

Finally, a brief note may be served on the unconventional treatment approach. Traditional Chinese medicine (TCM) is one of the cultural treasures of Chinese people and it is based on drugs traditionally composed of many different herbs with known or unknown active ingredients that can target various pathways for a given class of medical indications [85]. Chen et al. showed that the administration of Fufang Huangqi Decoction in eight MG patients (Myasthenia Gravis Foundation of America classes I and II) reduced the abundance of Blautia and Bacteroides and increased the presence of Bifidobacterium and Lactobacillus. These changes alleviated the symptoms of MG patients [71]. Although TCM originally appeared as a mythological doctrine, recently it has growing acceptance in Europe and in other Western countries [86]. Nevertheless, the use of herb-based food-supplements has become globally accepted in some diseases including autoimmune ones. However, the use of TCM in MG seems to be anecdotic; to date it remains a not-evidence based approach.

## 6. Conclusions

Oral and gut dysbiosis have found to be common in MG patients although their role in MG pathogenesis is unclear, having a direct or an indirect effect. Accordingly, dysbiosis may serve as a promoting factor for the development of comorbidities that may aggravate the MG course. Thus, several hypotheses may be raised. A first hypothesis points out molecular mimicry as a pathogenetic mechanism through which dysbiosis induces MG because of the reported case of MG onset after *Leptospira interrogans* acute infection [87]. In this view, the re-arrangement of intestinal flora would result in molecular mimicry phenomena underlying MG pathogenesis. However, there is not enough evidence to support this first hypothesis; therefore, it did not receive particular attention throughout our review. On the other hand, we focused mainly on the immunoregulatory function of microbiota and the effects of dysbiosis on the immune system. Accordingly, a possible link between dysbiosis and MG is associated with the reduction in *Clostridia* genera in gut microbiota and the subsequent alteration of SCFA production. This could result in the reduction in Foxp3+ CD4+ Tregs having a role in modulating the amounts of AChR antibodies [17]. In addition, the oral microbiota perturbations may aggravate the pro-inflammatory ambient in MG. The implications of oral and gut microbiota in MG pathogenesis are summarized in Figure 1. Although the perturbation of gut microbiota in patients with MG goes toward the creation of an inflammatory microenvironment, the exact molecular mediators and the timing of their involvement in the pathogenetic mechanisms of MG are still unknown. Thus, further studies appear necessary. In particular, the improvement of phylogenetic microarrays could allow to better understand the variations of the bacterial population in health and disease [2]. The F/B ratio represents a marker of gastrointestinal inflammation, and the finding are apparently conflicting in MG because of the *Firmicutes* reduction in gut microbiota and their increase in oral flora. The conflicting results between oral and gut microbiota deserve further investigation. However, since the gastrointestinal tract is widely populated by different bacterial species coming from the oral cavity, microbiota analyses must consider the large variety of the gastrointestinal bacterial flora in the various tracts [65]. A final consideration should be reserved for the comparison of gut microbiota alterations between MG and other autoimmune diseases. Indeed, several reports suggest that gut microbiota alterations in MG are shared with MS. The reduction in *Clostridia* and *Lactobacilli* is a common finding in both diseases, while some relevant differences need to be carefully examined and investigated to highlight their significance in both diseases [60]. In fact, an inverse relationship concerning the *Firmicutes* and *Bacteroidetes* phyla (e.g., Firmicutes are described increase in MS and reduced in MG, while Bacteroidetes seem to be reduced in MS and to increase in MG) is reported in some studies, although the common feature is the pro-inflammatory shift of the immune system. A possible explanation could be the specific and different variation of some genera within the two phyla. However, the comparison between MG and MS dysbiosis is limited because of the existence of more numerous studies on the microbiota in MS than in MG. The evidence on the microbiota involvement in MS and the increasing evidence of its involvement in MG pathogenesis could be the starting point for future studies. We supposed that microbiota may share a role in the pathogenesis of MG although, it is also reasonable that oral and gut microbiota alterations may be an indirect effect of MG because patients take a higher number of medications such as PPI immunosuppressants, pyridostigmine and corticosteroids which overall may alter microbiota composition. Additionally, the alteration of nutritional lifestyle and physical activity in patients with MG may have a further role in the microbiota perturbation. Thus, further studies are needed to clarify the direction of this close relationship between MG and microbiota alteration.

In conclusion, the alterations of oral and gut microbiota are intriguing factors in MG pathogenesis and disease severity although more studies are needed. First of all, it is necessary to increase the number of subjects analyzed and also to further extend the investigations on salivary microorganisms. In fact, to date, only one study is available that has investigated the oral bacterial composition. Moreover, the effects of oral hygiene and diet regimen on the oral and gut microbiota and MG severity need to be further investigated.

## Figures and Tables

**Table 1:** Human microbiota changes in patients with myasthenia gravis.

Gut Microbiota *	Changes	N. of MG Patients	Control Group	Significance	Reference
**Bacillota**					
Blautia	Increase	8 eating FHD	8 MG patients not eating FHD	*p* > 0.05	[71]
**Bacteroidetes**	Increase	70	74 HCs	*p* < 0.01	[72]
Bacteroides	Increase	10	10 HCs	*p* < 0.05	[39]
**Actinobacteria**	Reduction	10	10 HCs	*p* < 0.05	[39]
Bifidobacterium	Reduction	10	10 HCs	*p* > 0.05	[39]
**Firmicutes**	Reduction	52	49 HCs	*p* < 0.001	[37]
Clostridium	Reduction	52	49 HCs	*p* < 0.001	[37]
Eubacterium	Reduction	52	49 HCs	*p* < 0.001	[37]
Streptococcus	Increase	52	49 HCs	*p* < 0.001	[37]
Parasutterella	Increase	52	49 HCs	*p* < 0.001	[37]
Faecalibacterium	Increase	41	18 PD patients	*p* < 0.05	[40]
Lactobacillus	Reduction	NA	NA	NA	[73]
Roseburia	Reduction	8 eating FHD	8 MG patients not eating FHD	*p* < 0.05	[71]
**Proteobacteria**	Increase	52	49 HCs	*p* = 0.003	[37]
**Thermodesulfobacteriota**					
Desulfovibrio	Increase	10	10 HCs	*p* = 0.015	[39]
**Verrucomicrobiota**					
Verrucomicrobiaceae	Reduction	10	10 HCs	*p* < 0.05	[39]
Oral Microbiota *	**Changes**	**No. MG patients**	**Control group**	**Significance**	**Reference**
**Firmicutes**	Increase	20	20 HCs	*p* < 0.05	[65]
Streptococcus	Increase	20	20 HCs	*p* < 0.05	[65]
Oribacterium	Increase	20	20 HCs	*p* < 0.05	[65]
**Spirochaetota**	Reduction	20	20 HCs	*p* < 0.05	[65]
Treponema	Reduction	20	20 HCs	*p* < 0.05	[65]
**Proteobacteria**	Reduction	20	20 HCs	*p* < 0.05	[65]
Haemophilus	Reduction	20	20 HCs	*p* < 0.05	[65]
Neisseria	Reduction	20	20 HCs	*p* < 0.05	[65]
**Actinobacteria**					
Rothia	Increase	20	20 HCs	*p* < 0.05	[65]
**Bacillota**					
Lachnoanearobaculum	Increase	20	20 HCs	*p* < 0.05	[65]

Abbreviations: N, number; FHD, Fufang Huangqi Decoction; HCs, healthy controls. * Phyla are highlighted in bold while genus are listed under phyla category.

## Data Availability

Not applicable.
